# Prevalence of Attention Deficit Hyperactivity Disorder Among Children in Makkah Region, Saudi Arabia

**DOI:** 10.7759/cureus.35967

**Published:** 2023-03-10

**Authors:** Ziyad S Al-Saedi, Abdulrahman M Alharbi, Abdulkareem M Nmnkany, Bandar K Alzubaidi, Abed N Alansari, Mohammed Alhuzali, Mokhtar M Shatla

**Affiliations:** 1 Medicine, Centers for Disease Control and Prevention, Makkah, SAU; 2 Faculty of Medicine, Umm Al-Qura University, Makkah, SAU; 3 Faculty of Medicine, Ibn Sina National College, Jeddah, SAU; 4 Family Medicine, Faculty of Medicine, Umm Alqura University, Makkah, SAU

**Keywords:** children, saudi arabia, makkah region, prevalence, attention deficit hyperactivity disorder (adhd)

## Abstract

Introduction

Attention-deficit/hyperactivity disorder (ADHD) is the most common behavioral disorder in children and is described as a disease involving loss of self-control. The core symptoms of ADHD are inattentiveness, impulsivity, and motor unrest. Furthermore, poor concentration, distraction, hyperactivity, and poor academic achievement at school or at home are other symptoms. ADHD, like other prevalent medical disorders such as asthma and schizophrenia, may be impacted by several genes and has multiple contributing causes that are not all related to each other. The management of ADHD contains multimodal treatments, starting with psycho-education for parents and the child or adolescent patient in an age-appropriate manner called cognitive behavioral therapy. The worldwide prevalence rate of ADHD among children is 7.2% and, in some countries, can be higher and reach 15.5%; studies show the average prevalence of ADHD in the *Kingdom of Saudi Arabia* (KSA) is 9.2%.

Method

A descriptive cross-sectional study was conducted from December 2022 to January 2023 among children who live in Makkah between the ages of 4 and 14 years old via an online survey that contained the ADHD Rating Scale-IV for parents/teachers, and caregivers based on Diagnostic and Statistical Manual of Mental Disorders (DSM)-IV criteria. The scale was translated into Arabic and contains 18 questions about ADHD symptoms.

Result

The overall prevalence of combined ADHD among children in Makkah is 52.5% (n=203 out of 387); most of them were males (30.8%) and 21.7% were females (P=0.09), and most of the combined ADHD prevalence was between the ages of 11 and 14 (20.4%), then the age of 4-7 (16.8%), and 15.3% of them were between 8 and 10 years old. Additionally, the results show a higher prevalence of combined ADHD in Makkah city (33.1%) (n=121) in comparison to rural areas 19.4% (n=82) (P=0.132).

Conclusion

The aim of the study was to measure the prevalence rate of ADHD in the Makkah region. The study showed a high prevalence rate of ADHD (52.5%) among children in Makkah; the study was carried out online using an ADHD scale, and more accurate results could be found by using face-to-face interviews and including both parents and teachers.

## Introduction

Attention-deficit/hyperactivity disorder (ADHD), the most common behavioral disorder in children [1], is described as a disease involving a loss of self-control and is divided into three types: hyperactivity/impulsivity (ADHD-HI), inattention (ADHD-IA), and combined (ADHD-C). A child with ADHD may begin to exhibit symptoms before the age of 4 when there is a noticeable motor disturbance. Before this age, it can be difficult to distinguish an ADHD child from a non-ADHD one [2].

The core symptoms of ADHD are inattentiveness, impulsivity, and motor unrest [3]. Furthermore, poor concentration, distraction, hyperactivity, and poor academic achievement at school and at home are other symptoms [1]. Additionally, difficulties with speech and language, motor coordination, and the possibility of an autism spectrum disorder may be present. For some ADHD patients, their condition may be complicated by also having anxiety, depression, and, albeit less frequently, bipolar disorder. Additionally, ADHD symptoms and impairments may persist into adulthood and be linked to antisocial behavior, friendship problems, and poor performance at work [4].

ADHD, like other prevalent medical disorders such as asthma and schizophrenia, may be impacted by several genes and has multiple unrelated contributing causes [4]. Some studies consider it a familial disorder with a 5-9% relative risk in first-degree relatives [5]. However, while ADHD does share an inherited liability with other neurological and psychiatric problems such as autism spectrum disorder as well as developmental coordination problems related to intelligence quotient (IQ) and reading ability, genetic causes are not considered to be 100% of the cause of ADHD [4]. Indeed, environmental factors are also important because these are modifiable causes, including premature birth and in utero exposure to maternal neglect such as smoking cigarettes, consuming alcohol, and taking both illicit substances and prescribed drugs such as paracetamol [5].

The management of ADHD comprises multimodal treatments starting with psycho-education to parents as well as the child or adolescent patient in an age-appropriate manner through cognitive behavioral therapy, especially if they have mild symptoms or are under the age of six years [1,3]. Thereafter, treatment should continue if the patient still has moderate-to-severe ADHD or cognitive behavioral therapy fails [1].

The worldwide prevalence rate of ADHD among children is 7.2%, with it reaching 15.5% in some countries [6]. The average prevalence rate of ADHD in the KSA is 9.2% [7]. Although considerable research that measures the prevalence rate of ADHD has been published in cities such as Riyadh, Dammam, and Aseer [8-10], few studies have measured the prevalence rate of ADHD in a specific population in Makkah [11]. Further, no study has thus far measured the prevalence rate of ADHD among the general population of children in Makkah.

## Materials and methods

Study setting and population

A descriptive cross-sectional study was conducted from December 2022 to January 2023 among children between the ages of 4 and 14 years who live in Makkah through an online survey.

Sampling method

According to the general authority for statistics in Saudi Arabia, the number of children in the Makkah region is approximately 1,300,000. Hence, considering the 9.2% prevalence rate of ADHD in the KSA [7] and keeping the confidence interval at 95%, the required sample size was calculated to be 383 participants.

Data collection

Data were collected using a self-administered online questionnaire on Google Forms, completed by parents who have children between the ages of 4 and 14 years. The form contained two sections. The first section collected demographic data, including the child’s age (4-7, 8-10, 11-14 years) and gender, where the child lives (Makkah city or rural area), and current academic level (pre-school, primary school, secondary school). The second section contained the ADHD rating scale-IV for parents/teachers and caregivers based on the DSM-IV criteria. The scale was translated into Arabic and contained 18 questions about ADHD-HI and ADHD-AI symptoms. The odd numbers of items in the scale represented the IA type, and the even numbers represented the HI type. The answers to each question were provided on a four-point scale from 0 to 3 according to ADHD symptoms: 0, never/rarely; 1, sometimes; 2, often; and 3, very often. Both AI and HI were required to be present to diagnose the child with ADHD. The ADHD rating scale-IV has been validated and used in a previous study in Vietnam [12].

Ethical considerations

All participants provided informed consent before contributing to this study and were aware that their personal information would be kept anonymous and protected. Ethical approval for this study was provided by the Biomedical Research Ethics Committee of the Faculty of Medicine, Umm Al-Qura University (Approval No. HAPO-02-K-012-2022-11-1341).

## Results

Statistical software, IBM SPSS version 27 (SPSS, Inc., Chicago, IL), was used to analyze the data. The statistics were presented using numbers, percentages, means, and standard deviations. The connection between variables was evaluated using the chi-square test, with a level of significance of p-value < 0.05 and a confidence interval of 95%. A total of 400 participants completed the questionnaire; 13 were excluded for refusing consent at the beginning of the questionnaire, and the final number of participants was 387.

Out of the 387 participants, 244 (63%) were boys and 143 (37%) were girls. Most of them were aged 11-14 years (n=142, 36.7%), and the minority of them were aged 8-10 years (n=110, 28.4%). Furthermore, 244 (63%) of the participants were from Makkah city, and 143 (37%) of them lived in rural areas. Moreover, the majority were students at primary school (n=188, 48.6%), followed by secondary school (n=105, 27.1%), and pre-school (n=94, 24.3%) (Table 1).

**Table 1:** Demographic data

Percent	Frequency	Variable	
63	244	Male	Gender
37	143	Female	
34.9	135	4–7	Age
28.4	110	8–10	
36.7	142	11–14	
63	244	Makkah	Residency
37	143	Rural area	
24.3	94	Pre-school	Education level
48.6	188	Primary	
27.1	105	Secondary	

**Figure 1:**
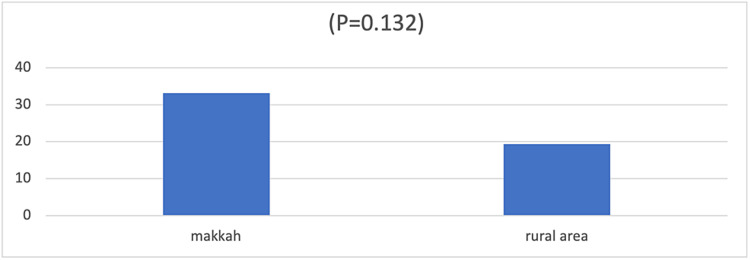
Comparison of attention-deficit/hyperactivity disorder prevalence between Makkah city and rural areas

The prevalence rate of ADHD-C among the children in Makkah was 52.5% (203 of 387), with a rate of 30.8% for boys and 21.7% for girls (P=0.09). The prevalence rate for those aged 11-14 years was 20.4%, followed by those aged 4-7 years (16.8%) and those aged 8-10 years (15.3%, P=0.459, Table 2). Additionally, the prevalence rate of ADHD in Makkah was 33.1% (n=121), compared with rural areas (19.4%, n=82, P=0.132, Figure 1). Primary school students had a higher prevalence rate of ADHD (24.9%, n=96) than secondary school students (14.2%, n=55) and pre-school children (13.4%, n=52, P=0.913, Table 3).

**Table 2:** Prevalence of attention-deficit/hyperactivity disorder between gender and age group

P-value	Percent		
0.09	30.8	Male	Gender
	21.7	Female	
0.459	16.8	4-7	Age
	15.3	8-10	
	20.4	11-14	

**Table 3:** Attention-deficit/hyperactivity disorder relating level of education

Education level	Frequency	Percent	P-value
Pre-school	52	13.4	0.913
Primary	96	24.9	
Secondary	55	14.2	

## Discussion

This study showed an increased prevalence of ADHD among children in the Makkah region (52.2%), with more males affected than females. Some studies have estimated that every classroom has at least one child suffering from this neurodevelopmental disorder [1,2,8]. Based on our comprehensive literature search, we found limited research on the prevalence rate of ADHD in the western region of the KSA [7]. Our study thus aimed to measure the prevalence rate of ADHD among children in Makkah, Saudi Arabia. It found that the prevalence rate of ADHD-C is 52.5%, which is significantly higher than that in other Saudi cities like Dammam (16.4%) and Al Riyadh (3.4%) [8,9]. This study showed that ADHD-C affects boys more than girls (30.8% boys, 21.7% girls). A previous study conducted in Riyadh showed a similar result [8]. In our study, ADHD-C was more prevalent in the age group of 11-14 years (20.4%), whereas other research in Dammam and the western region of Saudi Arabia has found that ADHD-C is more prevalent in the age group of 9-10 years [7,9]. We found that children who live in cities have a higher chance of being affected by ADHD than those in rural areas, and this point was made in other studies conducted in Vietnam [12]. In general, the worldwide prevalence rate of ADHD is 5.3-7.2%; however, geographical location may play a significant role in the explanation of this variability [13].

The increase in the prevalence rate of ADHD can affect social life and academic achievement at school for children, and we must increase awareness among families about this disease to diagnose it early and deal with it. The study has many limitations that might influence the interpretation of the findings. First, a larger study population would provide more significant results. Second, we used a self-administered online questionnaire translated from English into Arabic, which might have caused misunderstandings of the questions. Third, our study was used for screening purposes only, and this research was carried out as a college course requirement. Lastly, the questionnaire was distributed only to parents; instead, it should be completed by both parents and teachers.

## Conclusions

The aim of the study was to measure the prevalence rate of ADHD in the Makkah region. The study showed a high prevalence rate of ADHD (52.5%) among children in Makkah, with boys suffering more than girls. The study was carried out online using an ADHD scale, and more accurate results could be found by using face-to-face interviews and including both parents and teachers. We also recommend expanding the study across the Kingdom and increasing the sample size to get more accurate results on ADHD prevalence.
